# Effect of necrostatin-1 on sciatic nerve crush injury in rat models

**DOI:** 10.1186/s13018-023-03565-3

**Published:** 2023-01-30

**Authors:** Chen Yu, Xiaoxu Wang, Jian Qin

**Affiliations:** 1 Department of Orthopaedics, Sir Run Run Hospital, Nanjing Medical University, 109 Longmian Avenue, Nanjing, 210000 Jiangsu China; 2 Department of Orthopaedics, The Second Hospital, University of South China, Hengyang, 421000 Hunan China

**Keywords:** Necrostatin-1, Sciatic nerve injury, Necroptosis

## Abstract

**Background:**

Necrostatin-1 (Nec-1) is an inhibitor of the receptor interacting protein (RIP)1 kinase, which acts as an inhibitor of necroptosis, a special form of necrosis. In the present study, the effect of Nec-1 on peripheral nerve injury (PNI) was investigated.

**Methods:**

The PNI model was established by inducing sciatic nerve injury. Hematoxylin–eosin and immunofluorescence staining techniques were used to assess the extent of injury to nerve fibers and necrosis of Schwann cells (SCs). Western blotting was performed to detect the expression of necroptosis-related factors (RIP1 and RIP3). The concentrations of tumor necrosis factor-α, interleukin (IL)-1β, IL-6, and the oxidative stress-related enzyme malondialdehyde (MDA) were determined to indicate the degree of inflammation and oxidative stress.

**Results:**

Nec-1 could decrease the degree of peripheral nerve lesions after PNI and protect SCs and axons by inhibiting necroptosis. Furthermore, Nec-1 could reduce necroptosis by inhibiting RIP1 and effectively reduce inflammation and reactive oxygen species production at the early stage of PNI.

**Conclusions:**

Alleviation of necroptosis by Nec-1 may provide new insights into therapies for the early stages of peripheral nerve repair after PNI.

## Background

Peripheral nerve injury (PNI), a common disease in medical clinics, severely affects the quality of life of patients and increases their social burden [1]. More than one million patients are newly diagnosed with PNI worldwide every year [2]. Although injured peripheral nerves are capable of regeneration, the slow rate of axon regeneration and poor functional recovery are attributed to a decrease in nerve function in patients. However, rehabilitation of peripheral nerve function is mainly inhibited by posttraumatic inflammation and oxidative stress [3–5]. Control of reactive oxygen species (ROS) and suppression of post-traumatic inflammation can prevent further secondary injuries, which are very important in the early treatment of PNI. Peripheral nerves play vital roles in both motor and sensory nervous systems [6]. PNI can cause a variety of disorders such as permanent denervation, neuropathic pain, sensory disruptions, and movement limitations [7]. Nevertheless, the pathophysiological mechanisms underlying PNI are not fully understood, and there are no effective drugs or satisfactory therapies for the treatment of PNI [8, 9]. Exploring novel effective therapies for the treatment of PNI is of great significance. In recent years, PNI treatment has developed rapidly with the development of nerve transplantation and gene-and cell-based therapies [10, 11]. Interest in SCs has led to growing attention in the study of neural cell protection and reduction of cell death in the early stage of PNI.

Receptor-interacting protein (RIP)1 is a serine/threonine kinase that participates in both necroptosis and apoptosis. Necrostatin-1 (Nec-1) is an inhibitor of RIP1 [12], which is a key intermediate protein involved in the activation of necroptosis [13]. Necroptosis is a form of regulated cell death [14]. As well as being an inhibitor of RIP1, Nec-1 is also involved in necroptosis through inhibition of other relative factors. Previous studies have focused on the role of Nec-1 in the inhibition of pathological death in the central nervous system rather than in the peripheral nervous system (PNS). The role of Nec-1 in ischemic brain injuries and neurodegenerative diseases has been well studied [15, 16]; however, its effects on PNI remain unclear. Neuroprotection of Nec-1 reminds us that it may be a novel target for the treatment of PNI, our research aims to explore the function of Nec-1 in PNI.

## Materials and methods

### Animals and grouping

A total of 45 male Sprague–Dawley (SD) rats (aged 8–10 weeks and weighing 190–220 g) were used for surgical intervention. During the experimental procedure, one rat was housed per cage and maintained under a 12-h light/dark cycle with food and water ad libitum. All rats were divided randomly into sham operation (Sham group), PNI (PNI group), and PNI plus Nec-1 treatment (Nec-1 + PNI group) groups (*n* = 15 in each group). All the experiments were approved by the medical ethics committee of the University of South China.

### Experimental design

#### PNI model

To ensure that the compression force was consistent in the present neural injury model, all the operations were performed by the same researcher. SD rats were anesthetized with sodium pentobarbital (intraperitoneal; 40 mg/kg BW). The skin was cut to expose the sciatic nerve (1.0 cm distal to the sciatic notch). The nerve was crushed at a distance of 5 mm from the sciatic notch (midthigh) for 30 s. This process was repeated three times with crushed, clamped, and unclamped for 10 s using 14-cm hemostatic forceps at a locking tension of three [17] The overlying muscles and skin were sutured to each layer using 4–0 silk sutures and staples. After the operation, electrophysiological examination showed that there was no obvious action potential after the stimulation of the sciatic nerve, which proved that the model was successful. The rats were allowed to recover from the surgery on a 30 °C heating pad. Penicillin (40,000 U/day) was administered to prevent infection.

*Sham operation.* SD rats were anesthetized with sodium pentobarbital (intraperitoneal; 40 mg/kg body weight). After anesthesia, the skin of the SD rats was cut, and the sciatic nerves were isolated.

All rats in both groups exhibited no signs of peritonitis, pain, or discomfort following the intraperitoneal administration of sodium pentobarbital.

### Methods

The rats in the Nec-1 + PNI group received 1.65 mg/kg Nec-1 pretreatment (intraperitoneal; 0.05 ml/mg, 1 mg Nec-1 was dissolved in 0.05 ml DMSO, then diluted with saline to 1 ml; Med-ChemExpress) 15 min before the injury [18–20]. Rats in the sham and PNI groups received sham solvent treatment without Nec-1 following the same protocol. After the surgery, the rats were caged in a warm environment for 24 h and given clean water and food. PI (propidium iodide) labeling was used to distinguish between surviving early cells and necrotic cells. The specific methods are as follows: Rats were anesthetized as aforementioned. Subsequently, 1 ml PI (10 mg/ml, dissolved in DMSO and saline of equal volume) was used for in vivo labeling by injecting the tracer through each tail vein. After injection, the label was allowed to circulate for 1 h. Following euthanasia, the sciatic nerves were dissected (24 h after modeling), followed by hematoxylin–eosin (HE) staining, immunofluorescence experiments, western blotting, thiobarbituric acid (TBA) assays, and ELISA (3 rats per group for each methods) [21]. These techniques were used to detect the degree of sciatic nerve injury, the expression of S100 [a Schwann cell (SC) marker] and neurofilament (NF)200 (a neuronal marker), the degree of necrosis in SCs, the expression of necroptosis-related factors (RIP1 and RIP3), the level of posttraumatic inflammation, and the degree of oxidative stress after PNI. At the end of the experiment, the rats were euthanized with an overdose of sodium pentobarbital (intraperitoneal injection at a dose of 100 mg/kg BW). Death was confirmed by observing respiration and heartbeat and by checking the pupil and nerve reflex.

### HE staining

**Fig. 1:**
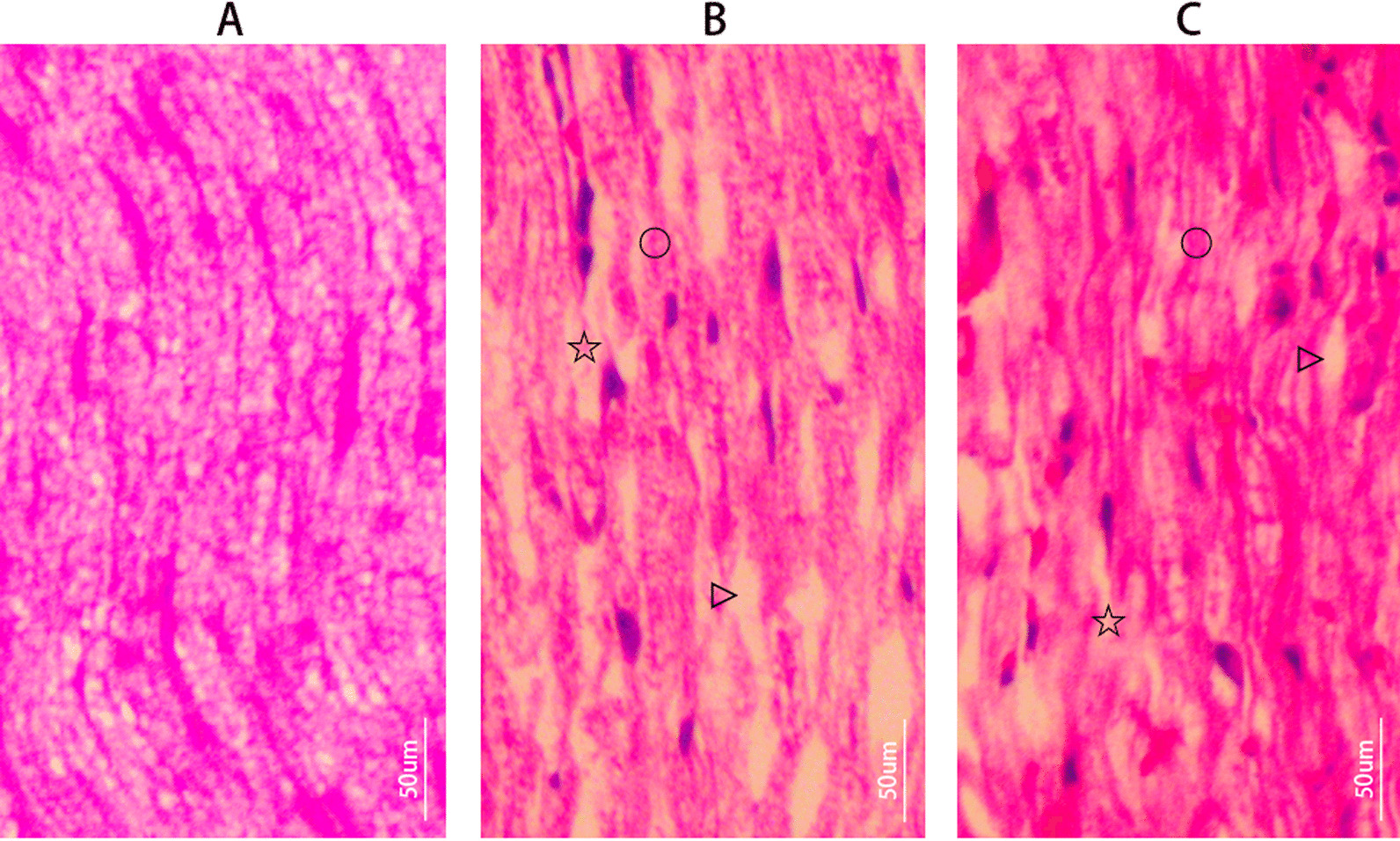
HE staining. **A** Sham group; **B** PNI group and **C** Nec-1 + PNI group. Pictures were taken using microscope (Olympus CX31, Japan, 20 ×, Scale bar = 50 um). (Circle) Swollen axon, (Star) Defective axon, (Right pointing triangle) Vacuolization

Four-micrometer longitudinal sections of injured sciatic nerve tissue at 24 h after PNI in each group (Sham, PNI, Nec-1 + PNI) were deparaffinized and stained in Harris hematoxylin solution for 15 min. After that, the sections were washed in running tap water for 1–3 s and differentiated in 1% acid alcohol for 1 min then washed briefly in ddH2O. The sections were stained in Eosin–phloxine solution for 3 min, dehydrated through two changes of 95% alcohol, 5 s each, two changes of 100% alcohol, 5 min each, and cleared in their changes of xylene, 3 min each. Finally, the sections were mounted with neutral balsam. Pictures were taken using an Olympus CX31 biological microscope and are shown in Fig. 1 (Longitudinal sections, 20 ×).

### Immunofluorescent staining

The Frozen cross sections (4 µm) were removed from the freezer and placed in an oven at 37 °C for 30 min for antigen retrieval, and all the sections were blocked with 10% normal serum blocking solution species containing 3% BSA, 0.1% Triton X-100 for 2 h at room temperature to avoid non-specific staining. They were then incubated with both primary antibodies at 4 °C overnight for different cell markers as follows: S100 (Schwann cell marker, Bioss, Beijing, China) and NF200 (neurofilament marker, Bioss, Beijing, China), washed with PBS, and incubated with secondary antibody (Yeasen, Shanghai, China) for 1 h at room temperature, washed twice with PBS for 5 min each, incubated with PI (Yeasen, Shanghai, China) for 30 min, washed twice with PBS for 5 min each time, and counterstained with nuclear counterstain 4,6-diamidino-2-phenylindole (DAPI, Yeasen, Shanghai, China). The stained sections were observed under a biological microscope (CX31, Olympus, Japan, 40 ×).

### Western blot

Western blotting was used for quantitative measurement of necroptosis-related factors. Total protein was extracted from injured sciatic nerve tissue of Sham, PNI and Nec-1 + PNI groups by RIPA protein lysis buffer (Beyotime, Shanghai, China) on cold ice (4 °C) for 30 min. Then the protein was collected and centrifuged (12,000 × g) at 4 °C for 20 min. The supernatant was collected and the protein concentration of the lysates was measured by the Protein BCA Assay Kit (Bio-Rad, Hercules, CA, USA). For the western blotting assay, 30 μg of protein mixed with 5 × SDS loading buffer was loaded per lane, separated with 12.5% SDS-PAGE and transferred to a polyvinylidene fluoride (PVDF) membrane (MilliporeSigma, Burlington, USA). After blocking in 5% fat-free milk for 2 h, the membranes were incubated with primary antibodies diluted in 5% fat-free milk overnight at 4 °C. The antibodies used were anti-RIP1 (Abcam,1 Kendall Square, Suite B2304 Cambridge, MA 02139-1517, USA), anti-RIP3 (Abcam,1 Kendall Square, Suite B2304 Cambridge, MA 02139-1517 USA), and anti-GAPDH (Bioworld Technology, Inc. 1660 South Highway 100, Suite 500 St. Louis Park, MN 55416, USA). After washing with 1 × PBS three times, the membrane was incubated with secondary antibody (Yeasen, Shanghai, China) for 1 h at room temperature and washed with 1 × PBS for 5 min three times. The membranes were visualized using enhanced chemiluminescence reagent (Beyotime, Shanghai, China). The relative intensity of each band was quantified using the ImageJ software from the National Institutes of Health (NIH).

### ELISA and MDA

After centrifugation at 1000 × g for 10 min, the supernatants of injured sciatic nerve tissue homogenates from the three groups were used to measure the concentrations of cytokines TNF-α, IL-1β, and IL-6 24 h after injury, which were detected using enzyme-linked immunosorbent assay (ELISA) kits (Biohj, Xiamen, Fujian, China) following the manufacturer's instructions. The results are expressed as nanograms per milligram of tissue (ng/mg tissue). The oxidative stress-related enzyme malondialdehyde (MDA) was detected in the supernatant of the lesion-site homogenate after centrifugation at 1000 × g for 10 min. The changes in MDA concentration in the three groups 24 h after injury were measured by the xanthine oxidase and thiobarbituric acid (TBA) methods, respectively, following the protocol provided by the detection kits (Njjcbio, Nanjing, Jiangsu, China). The MDA value was nmol/mg protein.

### Statistical analysis

Data are presented as mean ± SD. One-way ANOVA was used to compare multi-group variables, LSD test was used to compare inter-group variables. Statistical significance was set at *p* < 0.05.

## Results

### HE staining

HE staining showed that the nerve fibers in Sham group were continuous, orderly and compact, slightly wavy. The distal fibers of injured sciatic nerves in the PNI group and the Nec-1 + PNI group showed various degrees of swelling or defects. In the PNI group, the structure of nerve fibers was disordered, uneven, axons swelled, some axons disappeared, vacuoles formed, and fiber bundles were sparse. The Nec-1 + PNI group was less severe and had more compact fibers than the PNI group. Pictures in longitudinal sections were taken using microscope (Olympus CX31, Japan) and shown in Fig. 1 (20 ×, Scale bar = 50 um).

### Immunofluorescence staining

**Fig. 2:**
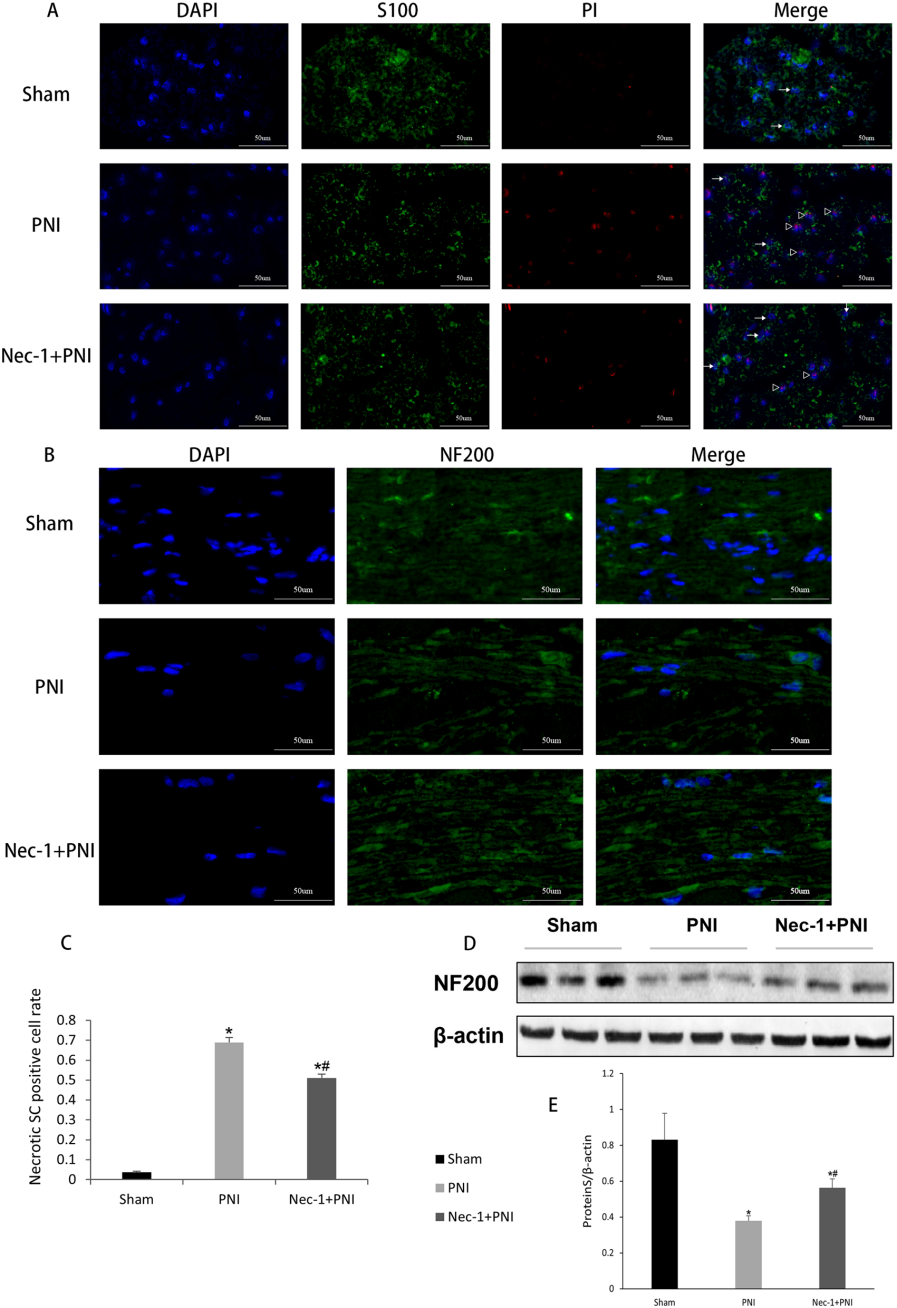
Immunofluorescence results of S100 (**A**) and NF200 (**B**) in sciatic nerve. Pictures were taken using microscope (Olympus CX31, Japan, 40 ×, Scale bar = 50 um). (Right pointing triangle) S100 and PI fluorescent colocalization cell (necrotic SC), (Rightwards arrow) S100 positive cell. **C** Necrotic SC positive cell rate in the lesion site of sciatic nerve in three groups at 24 h after PNI. **D** Western blot analysis was performed to study the expression of NF200 in uninjured adult rat sciatic nerve (Sham) and 24 h after a crush lesion (PNI, Nec-1 + PNI). **E** Semiquantitative analysis (relative density) of the intensity of NF200 to β-actin for each group. The data are mean ± SD (*p < 0.05, comparing the PNI or Nec-1 + PNI group with the Sham group; ^#^*p* < 0.05, comparing the Nec-1 + PNI group with the PNI group)

From immunofluorescence staining, S100 showed green fluorescence, DAPI-stained nuclei appeared blue, and PI staining showed red fluorescence. S100 green and PI red fluorescence appeared with colocalization, indicating the occurrence of necrosis in SCs. As shown in Fig. 2A, PI labeling showed that PI-positive cells were visible in sciatic nerves 24 h after injury. Compared with the Sham group, S100 and PI fluorescent colocalization cells were seen in the PNI group and Nec-1 + PNI group, indicating the necrosis of SCs in the early stage of PNI. As shown in Fig. 2A and Fig. 2C, S100 and PI fluorescent colocalization cells decreased in the Nec-1 + PNI group compared with the PNI group, indicating the necrosis of SCs in the Nec-1 + PNI group was less than in the PNI group (*p* < 0.05, One-way ANOVA and LSD test). This is more conducive to the regeneration and repair of peripheral nerve myelin sheath. Pictures were taken using microscope (Olympus CX31, Japan) and shown in Fig. 2A (40 ×, Scale bar = 50 um).

As shown in Fig. 2B, NF200 showed green fluorescence, and DAPI-stained nuclei were blue in immunofluorescence. At 24 h after injury, NF200 staining in the nerve fibers of the PNI and Nec-1 + PNI groups was less abundant and more parse than that in the sham group, indicating axonal disintegration of neuronal necrosis. Fiber bundles in the PNI group were more sparse. As shown in Fig. 2D,E, NF200 protein expression in the Nec-1 + PNI group was higher than that in the PNI group (*p* < 0.05, One-way ANOVA and LSD test), indicating that more survived neuronal axons in the Nec-1 + PNI group. Thus, our results demonstrated the protection of Nec-1 for neuronal axons. Pictures in longitudinal sections were taken using microscope (Olympus CX31, Japan) and shown in Fig. 2B (40 ×, Scale bar = 50 um).

### Analysis of necroptosis-related factors

**Fig. 3:**
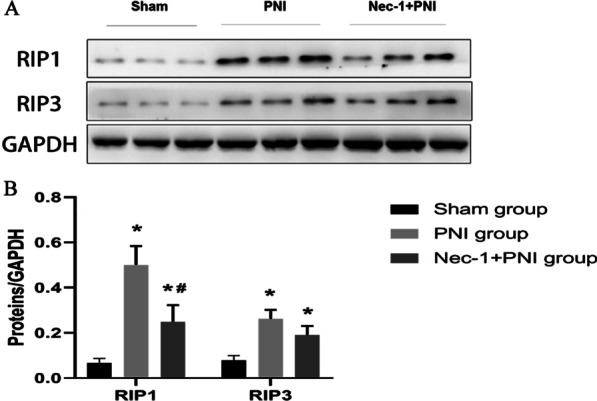
**A** Western blot analysis was performed to study the expression of RIP1, RIP3 in uninjured adult rat sciatic nerve (Sham) and 24 h after a crush lesion (PNI, Nec-1 + PNI). **B** Semiquantitative analysis (relative density) of the intensity of RIP1, RIP3 to GAPDH for each group. The data are mean ± SD (*p < 0.05, comparing the PNI or Nec-1 + PNI group with the Sham group; ^#^*p* < 0.05, comparing the Nec-1 + PNI group with the PNI group)

Western blotting was used to detect necroptosis-related factors at 24 h after PNI. Compared with the sham group, the protein expression levels of RIP1 and RIP3 were higher in the PNI and Nec-1 + PNI groups, and the expression of RIP1 in the Nec-1 + PNI group was lower than that in the PNI group (*p* < 0.05, One-way ANOVA and LSD test, Fig. 3). Although the expression of RIP3 in the Nec-1 + PNI group was lower than that in the PNI group, the difference was not statistically significant. These data suggest that Nec-1 can alleviate cellular necroptosis during the early stage of PNI.

### Effects of Nec-1 on oxidative stress-related enzymes and cytokines

**Fig. 4:**
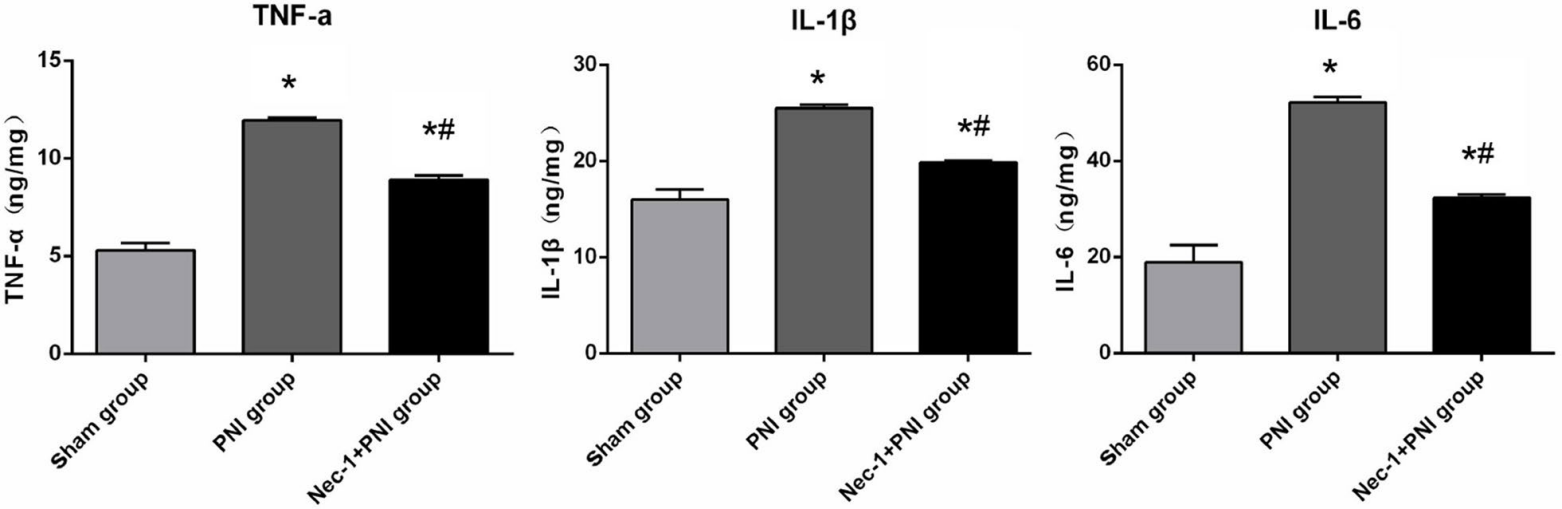
Cytokines detection for TNF-a (ng/mg tissue), IL-1β (ng/mg tissue) and IL-6 (ng/mg tissue) in the lesion site of sciatic nerve in three groups at 24 h after PNI. Data are presented as mean ± SD (*p < 0.05, comparing the PNI or Nec-1 + PNI group with the Sham group; ^#^*p* < 0.05, comparing the Nec-1 + PNI group with the PNI group)

**Fig. 5:**
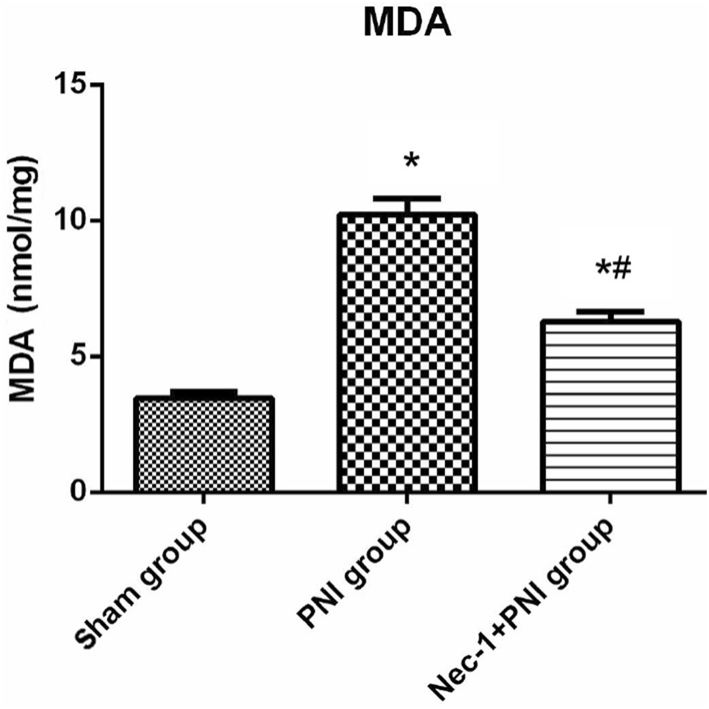
Oxidative stress-related enzymes detection. Concentration of MDA (nmol/mg protein) in three groups at 24 h after PNI. Data are presented as mean ± SD (*p < 0.05, comparing the PNI or Nec-1 + PNI group with the Sham group; ^#^*p* < 0.05, comparing the Nec-1 + PNI group with the PNI group)

Figure 4 shows a comparison of TNF-α, IL-1β, and IL-6 concentrations at the lesion site among the three groups; the levels of these cytokines in the sham group were lower than those in the PNI and Nec-1 + PNI groups. After treatment with Nec-1, the levels of these cytokines were reduced in the Nec-1 + PNI group (*p* < 0.05, One-way ANOVA and LSD test). Figure 5 shows a comparison of MDA concentrations among the three groups. MDA levels were significantly higher in the PNI group than in the sham group. The MDA concentration was reduced after treatment with Nec-1, although it was still higher than that in the sham group (*p* < 0.05, One-way ANOVA and LSD test).

## Discussion

SCs, the principal glial cells in the PNS, not only promote PNS integrity [22] but also maintain the function and survival of PNS neurons [23]. Dysfunction of SCs can cause axon demyelination, disorders of axonal regeneration, directional guidance of axons [22, 24–27]; and neurodegenerative diseases [23]. In the acute stage of PNI, apoptosis and necrosis are the most common forms of SC death. Inhibition of apoptosis can partially protect SCs [28–31], and numerous studies have focused on the regulation of SC apoptosis. However, inhibition of necrosis may also protect SCs.

Few investigations have focused on the role of necrosis, which is an accidental cell death caused by extreme physiological, mechanical, or thermal stress [32]. The number of necrotic cells is determined by the severity of the injury, ischemia, edema, secondary inflammatory response, and other factors. Necroptosis and necrosis have similar morphological features, but necroptosis is a programmed form of necrotic cell death that can be regulated [33]. Using the same necroptosis and apoptosis pathways, necrotic cell death can be triggered through a complex consisting of RIPK1 and RIPK3 in the absence of caspase8 activity [34, 35]. Nec-1 was discovered as an inhibitor of this pathway, which inhibits the function of RIP1 through the necrotic kinase, RIP1/RIPK1 [12]. Several studies have disrupted necroptosis to investigate the neuroprotective effects of Nec-1 and have identified its protective potential in various models [13, 36–39]. However, little is known regarding the effects of PNI on necroptosis. Whether Nec-1 can boost cell protection during PNI remains unclear.

SC necrosis was observed in both the PNI and Nec-1 + PNI groups in the present study. Necrosis may lead to further damage to the peripheral nerve tissues after PNI. Sections of injured sciatic nerves with reduced vacuolization and necrosis were observed in the Nec-1 + PNI group compared with those in the PNI group. The present results showed that Nec-1 inhibited necrosis and protected nerves by preventing vacuolar, axonal, and myelin degeneration after PNI. RIP1 and RIP3 were downregulated in the Nec-1 + PNI group 24 h after PNI, indicating inhibition of necroptosis. The present study took samples 24 h after PNI, according to the results that SCs begin to dramatically decrease at this time [21]. To date, few studies have focused on the regulation of necroptosis to investigate the neuroprotective effect of Nec-1 in PNS. The present study explored the role of Nec-1 in PNS and found that inhibition of necroptosis may protect SCs and axons.

The functional recovery of peripheral nerves is obstructed by posttraumatic inflammation and oxidative stress [3–5]. Posttraumatic inflammation after PNI is also driven by various inflammatory factors such as TNF-a, IL-1β and IL-6 involved in nerve injury [40, 41]. Detection of cytokines at the lesion site is more accurate and convenient than detection in the serum for the prediction of inflammation after PNI; therefore, these cytokines were detected in the sciatic nerve tissue using ELISA kits. The results showed that TNF-α, IL-1β, and IL-6 levels at 24 h after injury were the lowest in the sham group, and the levels in the PNI group were higher than those in the Nec-1 + PNI group. Nec-1 has a positive effect on relieving inflammation; however, the underlying mechanism remains unclear. Whether Nec-1 inhibits cytokines directly or through inhibition of necrosis requires further investigation. Oxidative stress may also contribute to secondary injuries after PNI. SCs and macrophages express pro-inflammatory factors, leading to the expression of ROS at the injury site [42–44]. MDA is the main oxidative stress-related enzyme; therefore, the concentration of MDA was measured after PNI. MDA concentrations were higher in the PNI group than in the Nec-1 + PNI group, with the lowest levels observed in the sham group. These results show that Nec-1 modulates inflammatory factors through the inhibition of oxidative stress. Mitochondria are energy production and ATP synthesis organelles that provide the basis for oxidative stress formation and are also the main targets of oxidative insults, leading to disruption of the normal cell cycle [45, 46]. As such, it was speculated that maintaining mitochondrial function may provide new insights into the treatment of PNI with Nec-1. The effects of Nec-1 on myelin and axonal regeneration should be further investigated in future studies.

The major limitation of the present research is that the neural function (behavioral and electrophysiological observations) and time point results after the initial injury were not investigated. Detailed neural function studies and long-term follow-up studies should be conducted in the future.

The present study showed that Nec-1 could protect SCs and axons by inhibiting necroptosis, which is conducive to axonal regeneration and repair of peripheral nerve myelin. Therefore, Nec-1 may decrease the incidence of peripheral nerve lesions. The present findings also provide evidence that Nec-1 could reduce necroptosis by inhibiting RIP1 and RIP3 recruitment and effectively reduce inflammation and ROS production in the early stages of PNI. In conclusion, Nec-1 alleviated of necroptosis may provide new insights into early stage treatment of peripheral nerve repair after PNI.

## Data Availability

The analyzed datasets generated during the present study are available from the corresponding author upon reasonable request.

## Abbreviations

ELISA: Enzyme-linked immunosorbent assay
HE: Hematoxylin–eosin
IL-1β: Interleukin-1β
MDA: Malondialdehyde
Nec-1: Necrostatin-1
PNI: Peripheral nerve injury
PNS: Peripheral nervous system
PVDF: Polyvinylidene fluoride
ROS: Reactive oxygen species
RIP: Receptor-interacting protein
SCs: Schwann cells
TNF-α: Tumor necrosis factor-α
TBA: Thiobarbituric acid
